# Multimodal input for vocabulary learning: Chinese EFL learners’ perceived effectiveness across input combinations, word types, and proficiency levels

**DOI:** 10.3389/fpsyg.2026.1783303

**Published:** 2026-03-23

**Authors:** Junfan Liu, Haoyu Wang

**Affiliations:** 1 Faculty of Arts and Education, University of Auckland, Auckland, New Zealand; 2 Business School, University of Auckland, Auckland, New Zealand

**Keywords:** Chinese EFL learners, learner proficiency, multimodal input, perceived effectiveness, vocabulary learning, word type

## Abstract

This study investigated how Chinese learners of English perceive the effectiveness of different multimodal input for vocabulary learning. Forty participants perceived 14 combinations of visual, auditory, tactile, and gestural modalities across various word types and proficiency levels through an online questionnaire. Results revealed three key findings. First, adding more modalities did not automatically increase perceived effectiveness; what mattered was which specific modalities were combined. Visual-inclusive combinations consistently received the highest ratings, while gestural input alone was rated lowest. Second, learners showed distinct preferences for different word types: concrete words favored visual and auditory input, action words preferred visual and gestural input, and emotion words selected auditory and gestural input. Abstract words elicited no clear consensus. Third, proficiency influenced these patterns. Advanced learners rated multimodal input more favorably overall and showed convergence on visual and auditory combinations for abstract words, whereas intermediate learners remained divided and reported higher processing difficulty. These findings document systematic patterns in learner perceptions and provide a foundation for experimental studies testing whether such preferences correspond to actual vocabulary learning outcomes.

## 1 Introduction

How learners receive and process lexical information may influence learners’ perceptions of vocabulary learning effectiveness. Traditional classroom instruction has relied primarily on written text and spoken input, but multimedia technology now enables a broader range of options. Learners can engage with vocabulary through sight, sound, touch, and movement—combining these channels is known as multimodal input (Cárdenas-Claros et al., 2023). Such input is increasingly common in language learning contexts, from video-based instruction to gesture-integrated activities (Montero Perez, 2020).

Two theoretical frameworks generate differential predictions about the effectiveness of multimodal vocabulary input. Paivio and Walsh (1993) Dual Coding Theory (DCT) proposes that cognition operates through two independent but interconnected systems: a verbal system for language and a nonverbal system for mental imagery. When learners encounter information through both systems simultaneously—such as a visual image paired with spoken narration—dual encoding creates two retrieval pathways, increasing the probability of recall. This advantage is predicted to be strongest for concrete words, which have clear sensory referents and are more readily encoded as mental images, and for action words, where bodily movement may activate sensorimotor representations that support encoding (Kana et al., 2012; Macedonia, 2014). For abstract words, however, the nonverbal system offers fewer accessible images, which limits the dual-coding advantage (Borghi et al., 2017; Mayer, 2024).

Cognitive Load Theory (CLT) generates a partially competing prediction. Sweller et al. (2011) argue that working memory capacity is finite, and that the total cognitive load imposed by a learning task constrains what can be processed at any given time. When input channels increase from single to dual to triple modalities, the cumulative demands may exceed available capacity, particularly for learners with limited proficiency who lack the schemas needed to manage multiple information streams efficiently (Kalyuga, 2007). Under this account, adding modalities does not automatically improve learning and may reduce perceived effectiveness when cognitive resources are strained.

These frameworks yield differential predictions. DCT predicts that visual-inclusive combinations should be perceived as most effective, particularly for concrete and action words with clear imageable referents. CLT predicts that lower-proficiency learners—who lack the schemas to manage multiple information streams—may find complex combinations more cognitively demanding than advanced learners (Kalyuga, 2007), such that modality quantity alone does not determine perceived effectiveness. Mayer’s (2009) cognitive theory of multimedia learning similarly holds that visual and auditory channels operate with limited capacity, such that effective instruction distributes rather than saturates processing demands.

Empirical research offers partial support for both frameworks, though findings are distributed across studies that examined different modality pairings and populations. Bisson et al. (2014) exposed Welsh learners to target words through text, images, and audio, finding that even two trimodal exposures improved translation recognition relative to untreated words. Xing and Zhang (2025) compared visual-only, auditory-only, and audiovisual conditions among 90 Chinese learners and found no group differences on immediate tests, but the audiovisual condition produced significantly stronger retention on delayed posttests. These findings suggest that dual-channel benefits are not guaranteed by combining input types and may depend on the time available for consolidation.

Word type moderates these effects in systematic ways. Farley et al. (2014) found that visual input significantly improved abstract word recall but did not benefit concrete word recall, suggesting that concrete words may already carry sufficient visual-semantic associations. Huang et al. (2019) demonstrated that gestural input improved action verb recognition by 8–10%, consistent with embodied cognition accounts linking physical enactment to sensorimotor representation. Altarriba and Basnight-Brown (2012) examined how English speakers learned Spanish concrete, abstract, and emotion words through visual and auditory input: emotion words showed faster initial processing but lower translation accuracy and higher error rates under interference, which the authors attributed to the absence of affective context in decontextualized learning. Together, these findings indicate that word type systematically shapes which modality pairings support encoding.

Input sequence and proficiency represent two further dimensions. Yuan and Tang (2025) compared subtitle conditions among Chinese EFL learners and found that presenting L1 subtitles before bilingual subtitles outperformed other orders on both form and meaning recall, with the lowest associated cognitive load, *F*(3, 158) = 96.21, *p* < 0.001, partial *η*^2^ = 0.65. Yu and Liu (2022) similarly found that presenting pictures before L1 translations produced stronger retention than the reverse order on both immediate (Cohen’s *d* = 1.27) and delayed posttests (*d* = 0.73). Regarding proficiency, Ting (2013) found that approximately 70% of lower-proficiency learners relied on subtitles as necessary support, whereas 57% of upper-intermediate learners deliberately avoided them to reduce dependency—a pattern consistent with CLT’s prediction that more advanced learners process familiar input with lower cognitive cost (Kalyuga, 2007).

Chinese EFL learners constitute a theoretically meaningful population for examining these questions. English instruction in Chinese secondary and tertiary education has historically been organized around teacher-centered delivery, written vocabulary lists, and repetitive copying exercises, with comparatively greater emphasis on visual and written input, and limited systematic incorporation of auditory and gestural channels (Hu, 2002; Liu and Ren, 2024; Fu, 2021). This instructional pattern means that Chinese learners typically enter multimodal learning contexts with extended experience in visual and tactile input—specifically reading and handwriting—while auditory and gestural channels remain less institutionally established. According to CLT, familiarity with a channel reduces the extraneous load associated with processing it (Kalyuga, 2007), which suggests that Chinese learners may evaluate familiar modalities more favorably not because those modalities produce stronger learning, but because they impose lower processing demands. Mayer’s (2009) cognitive theory of multimedia learning was developed largely in Western educational contexts and assumes that learners bring relatively balanced experience across visual and auditory channels. Whether this assumption holds for learners whose instructional history has systematically emphasized particular modalities over others has not been empirically examined.

Despite these findings, several gaps remain. First, prior studies have examined specific modality pairings—primarily visual–auditory combinations or isolated single-modality comparisons—without systematically comparing all combinations of visual, auditory, tactile, and gestural input within the same study. Second, existing research has measured learning outcomes but has not documented how learners perceive the relative effectiveness of different combinations, or how these perceptions interact with word type and proficiency level. Learner perceptions matter because they influence strategy selection and engagement (Liaw and Huang, 2013), and divergences between perceived and actual effectiveness represent a concern for instructional design. Third, the extent to which Chinese learners’ distinct instructional background shapes modality preferences—and whether this pattern varies with proficiency—has not been directly investigated.

Given these gaps, this study examines Chinese EFL learners’ perceived effectiveness and cognitive load across four input channels: visual, auditory, gestural, and tactile. Gestural input refers to whole-body movements representing lexical meaning, such as acting out “jump” (Macedonia, 2014). Tactile input, operationally defined in this study, encompasses learning activities involving physical hand engagement with written materials, including handwriting, copying, and manipulating flashcards. Research has demonstrated that handwriting creates motor memory traces through graphomotor processes, which facilitate subsequent character recognition (Longcamp et al., 2008). While these activities vary in their specific motor patterns, they share the common feature of repetitive fine motor movements and haptic interaction with vocabulary materials—a pedagogical practice widely employed in Chinese EFL classrooms.

*RQ1*: Which input combinations do learners perceive as most effective for vocabulary learning, and how does input complexity relate to perceived effectiveness?

*RQ2*: What are the preferred patterns of different combinations for learning vocabulary categories, including combination types and presentation sequences?

*RQ3*: What differences exist in input preferences among learners at different proficiency levels?

These questions examine learner perceptions and preferences, which may inform instructional design while requiring empirical validation through performance-based studies.

## 2 Method

### 2.1 Participants

Forty Chinese EFL learners participated (36 female; ages 18 and above), categorized by proficiency based on recent standardized test scores where available, or by self-assessment otherwise: lower intermediate (IELTS below 6; *n* = 10), intermediate (IELTS 6.5–7.5; *n* = 20), and advanced (IELTS 8–9; *n* = 10). The questionnaire used the term “beginner” for lowest proficiency group; however, since IELTS below 6 corresponds approximately to CEFR B1–B2, “lower-intermediate” is used throughout this paper to avoid confusion with absolute beginners. Prior experience with input types varied: visual (*n* = 38), auditory (*n* = 38), tactile (*n* = 29), and gestural (*n* = 16). Such experience was not required as the questionnaire described each input type with concrete examples, though differential familiarity with modalities may have influenced ratings and should be considered a potential confound.

### 2.2 Materials

A Qualtrics questionnaire measured perceived effectiveness of multimodal input combinations across five sections. First, it collected participants’ basic demographic and language learning background information. Second, it assessed learners’ perceptions of the effectiveness of different multimodal input types (visual, auditory, tactile, gestural) and their combinations for vocabulary learning. Third, it explored processing challenges and cognitive load when using multiple input types simultaneously. Fourth, it examined how different word types (concrete, abstract, action, emotion words) interact with various input combinations. Fifth, it investigated learner preferences regarding input presentation sequence for different word types. Concrete examples were provided throughout the questionnaire to ensure participants understood each input type and combination.

No fixed vocabulary items were used as learning targets. Example words were included to help participants understand each word type category (e.g., *table* for concrete nouns, *run* for action verbs, *freedom* for abstract words, *joy* for emotion words), not as stimuli to be learned or recalled. Since participants rated their perceptions of input modalities at the category level, systematic control of word frequency or imageability was not required for the present purpose. That said, individual example words may have shaped how participants interpreted each category, and future studies would benefit from using multiple exemplars or validated word lists.

Face validity was established through iterative review of item wording by both authors to ensure adequate coverage of the target constructs. Content validity was supported by grounding item content in established theoretical definitions—for instance, gestural input was operationalized following Macedonia (2014), and cognitive load items were informed by Sweller et al. (2011). No formal pilot study was conducted prior to administration.

The questionnaire used 5-point Likert scales throughout. Perceived effectiveness items ranged from 1 (*Not effective at all*) to 5 (*Very effective*). Processing difficulty was assessed with five items rated from 1 (*Never*) to 5 (*Always*), covering information overload, processing time, attentional focus, input confusion, and mental fatigue. For input sequence preferences, participants selected from three ordering options (e.g., visual → auditory → gestural). For best combination questions, participants selected one option from six dual-modality combinations. The processing difficulty scale showed strong internal consistency (Cronbach’s *α* = 0.91, 95% *CI =* [0.87, 0.94]), with item-total correlations ranging from r = 0.74 to r = 0.87, indicating that all items contributed to a coherent measure of perceived cognitive load.

### 2.3 Procedure

Participants were recruited voluntarily via social media. After providing informed consent, participants completed an online questionnaire administered through Qualtrics. The survey took approximately 20–25 min. Participants indicated their English proficiency level based on their most recent test scores (e.g., IELTS) or self-assessment, then completed all sections in a fixed order.

### 2.4 Data analysis

All analyses were conducted in R using lme4 and lmerTest for mixed-effects modeling (Bates et al., 2015; Kuznetsova et al., 2017). Likert-scale responses (1–5) were treated as continuous, following evidence that parametric analyses are generally robust with such data (Norman, 2010).

For RQ1, a linear mixed-effects model (LMM) explored how perceived effectiveness ratings varied across 14 input combinations, with Complexity (single/dual/triple) and Proficiency as fixed effects and participants as random intercepts. Additionally, an exploratory analysis examined self-reported processing difficulty to explore factors potentially associated with proficiency differences. Processing difficulty was computed as the mean of five items assessing information overload, processing time, focus difficulty, confusion, and mental fatigue (Cronbach’s *α* = 0.91). While these items tap conceptually distinct aspects of processing demands, their high internal consistency (*α* = 0.91) suggests participants experienced them as a unified construct. A Pearson correlation assessed the association between perceived effectiveness and processing difficulty.

For RQ2, an LMM explored whether perceived effectiveness of multimodal input differed by Word Type (concrete/abstract/action/emotion) and Proficiency. Chi-square goodness-of-fit tests examined whether participants’ combination preferences deviated from uniform distributions within each word type. An omnibus chi-square test on the 4 × 6 contingency table assessed whether preference patterns differed across word categories. Participants’ preferred input sequences were similarly analyzed using.

chi-square tests. To address the risk of Type I error across multiple chi-square comparisons, Bonferroni correction was applied to two families of tests: proficiency-related combination preference tests (four word types; corrected *α* = 0.0125) and sequence-related tests (four comparisons; corrected *α* = 0.0125). Uncorrected *p*-values are also reported given the exploratory nature of the study.

For RQ3, proficiency was included as a fixed effect in all LMMs to explore potential differences among proficiency groups. Chi-square tests examined whether combination preferences and beliefs about sequence importance differed across proficiency levels. Given sparse cells in some crosstabulations, Monte Carlo simulated *p*-values supplemented asymptotic results.

For all LMMs, Type III F-tests used Satterthwaite approximation. Significant effects were followed by Tukey-adjusted pairwise comparisons. Partial *η*^2^ was computed to estimate effect sizes, with values of 0.01, 0.06, and 0.14 representing small, medium, and large effects (Cohen, 1988). Cramér’s *V* indexed association strength for chi-square tests, with 0.10, 0.30, and 0.50 as small, medium, and large effect benchmarks (Cohen, 1988).

## 3 Results

### 3.1 Perceived effectiveness of input combinations

**Figure 1:**
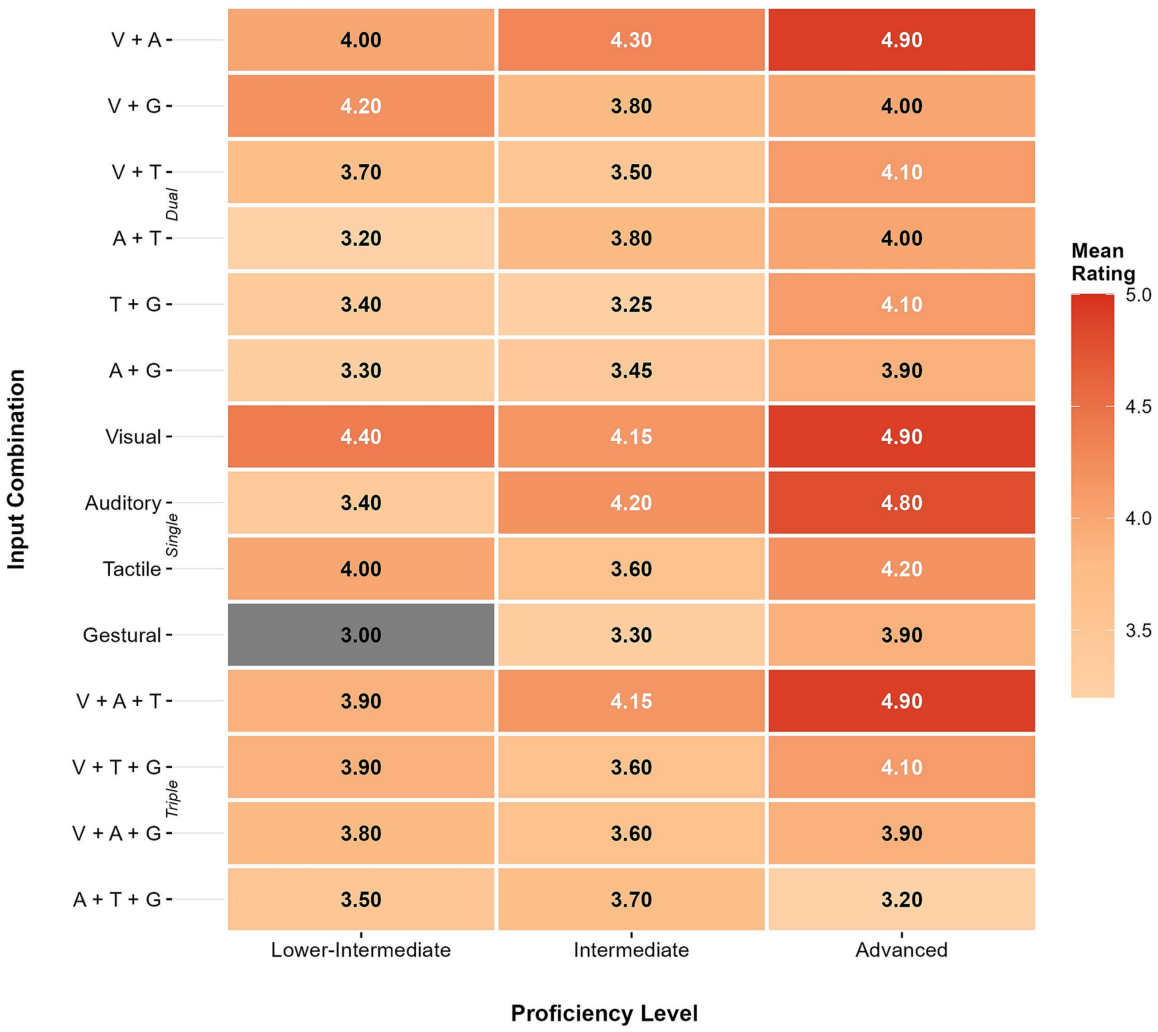
Heatmap of mean perceived effectiveness ratings by proficiency level and input combination complexity. Visual-based inputs (single visual and *V + A*) consistently received the highest scores, while triple combinations showed no clear advantage.

Participants rated the perceived effectiveness of 14 input combinations on a 5-point scale. Figure 1 presents mean ratings across proficiency levels. The three combinations receiving the highest perceived effectiveness ratings all included visual input: visual alone (*M* = 4.40, *SD* = 0.74), visual + auditory (*M* = 4.37, *SD* = 0.87), and visual + auditory + tactile (*M* = 4.28, *SD* = 0.72). Gestural alone received the lowest rating (*M* = 3.37, *SD* = 1.17).

A linear mixed-effects model with participants as random intercepts examined the effects of word type and proficiency level on perceived effectiveness ratings (Table 1). The main effect of complexity was not significant, *F*(2, 514) = 1.54, *p* = 0.216, *η*^2^ₚ = 0.01, a small effect, with estimated marginal means (EMMs) of 3.94, 3.79, and 3.83 for single, dual, and triple combinations. The complexity × proficiency interaction was also non-significant, *F*(4, 514) = 0.93, *p* = 0.446, *η*^2^ₚ = 0.01. However, the main effect of proficiency was significant, *F*(2, 38) = 7.99, *p* = 0.001, *η*^2^ₚ = 0.29, a large effect. Advanced learners (EMM = 4.21) gave higher ratings than intermediate (EMM = 3.75, *p* = 0.002) and lower-intermediate learners (EMM = 3.70, *p* = 0.004); the latter two groups did not differ (*p* = 0.919). To explore factors that might be associated with these proficiency differences in perceived effectiveness, we examined participants’ self-reported processing difficulty.

**Table 1:** Linear mixed model (LMM) analyses for perceived input effectiveness, helpfulness, and processing difficulty.

Analysis	Effect	*F*	*df*	*p*	*ηp^2^*
Input Perceived Effectiveness	Complexity (C)	1.54	(2, 514.0)	0.216	0.01
	Proficiency (P)	**7.99**	**(2, 38.4)**	**0.001**	**0.29**
	C × P	0.93	(4, 514.0)	0.446	0.01
Word type helpfulness	Word type (W)	**5.06**	**(3, 111.0)**	**0.003**	**0.12**
	Proficiency (P)	**11.63**	**(2, 37.0)**	**<0.001**	**0.39**
	W × P	**2.98**	**(6, 111.0)**	**0.010**	**0.14**
Perceived processing difficulty	Dimension (D)	1.91	(4, 148.0)	0.111	0.05
	Proficiency (P)	**22.18**	**(2, 37.0)**	**<0.001**	**0.55**
	D × P	1.57	(8, 148.0)	0.139	0.08

df, degrees of freedom; *ηp*^2^, partial eta squared. Significant effects (*p* < 0.05) are shown in bold. Proficiency Level yielded significant main effects across all measures, while a significant interaction between word type and proficiency (W × P) was observed for helpfulness ratings.

An exploratory LMM examining perceived processing difficulty revealed a significant main effect of.

Proficiency, *F*(2, 37) = 22.18, *p* < 0.001, *η*^2^_p_ = 0.55, a large effect by conventional benchmarks (Cohen, 1988). Advanced learners reported the lowest perceived difficulty (*M* = 2.26), followed by intermediate (*M* = 3.67) and lower-intermediate learners (*M* = 4.02). Perceived processing difficulty correlated negatively with perceived effectiveness, *r* = −0.48, *p* = 0.002, suggesting that learners who found multimodal input more demanding also rated it as less effective.

### 3.2 Word type perceived effects and proficiency interaction

Participants rated the perceived effectiveness of multimodal input for four word types (1 = *not helpful at all*, 5 = *very helpful*). A linear mixed-effects model with participants as random intercepts examined the effects of word type and proficiency level on perceived effectiveness ratings (Table 1).

The main effect of word type was significant, *F*(3, 111) = 5.06, *p* = 0.003, *η*^2^ₚ = 0.12, a medium effect.

Estimated marginal means were highest for concrete words (EMM = 4.42), followed by action words (EMM = 4.30), emotion words (EMM = 4.12), and abstract words (EMM = 3.85). Tukey-adjusted pairwise comparisons indicated that abstract words were rated significantly lower than concrete words (*p* = 0.002) and action words (*p* = 0.023); no other pairwise differences reached significance (*p*s > 0.22).

**Figure 2:**
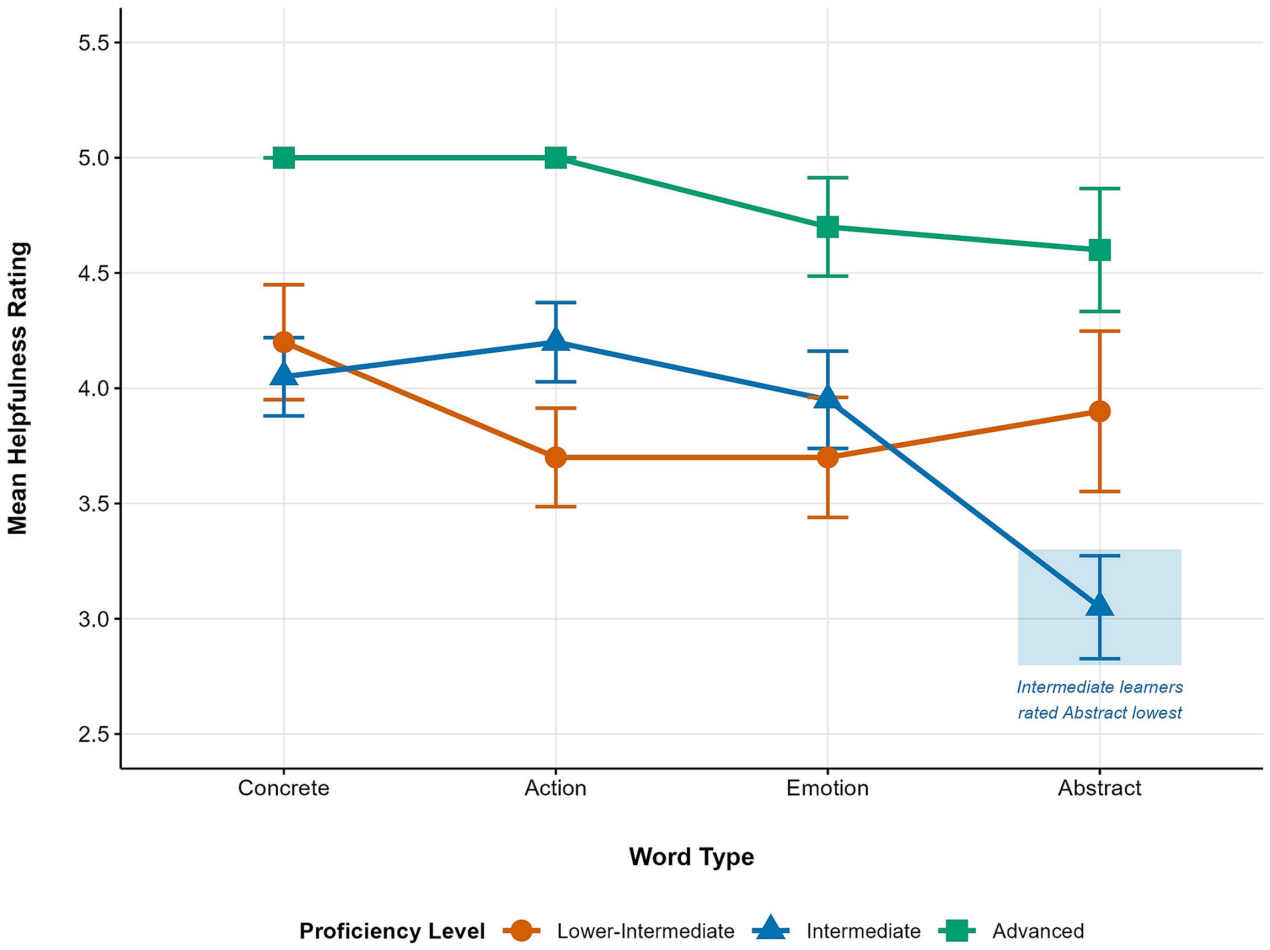
Interaction of proficiency level and word type on mean helpfulness ratings. A significant drop in ratings is observed for intermediate learners specifically in the abstract category.

The main effect of proficiency was also significant, *F*(2, 37) = 11.63, *p* < 0.001, *η*^2^ₚ = 0.39, a large effect, as was the word type × proficiency interaction, *F*(6, 111) = 2.98, *p* = 0.010, *η*^2^ₚ = 0.14, a large effect (Figure 2). Advanced learners gave the highest ratings across all word types (EMMs = 4.60–5.00). For concrete, action, and emotion words, lower-intermediate and intermediate learners showed similar ratings (EMMs = 3.70–4.20). Ratings for abstract words showed a U-shaped trend across proficiency levels: lower-intermediate (*M* = 3.90), intermediate (*M* = 3.05), and advanced (*M* = 4.60). Advanced learners rated abstract words significantly higher than both intermediate (*p* = 0.046) and lower-intermediate learners (*p* = 0.017), while the difference between lower-intermediate and intermediate learners was not significant (*p* = 0.70).

### 3.3 Preferred input combinations by word type

**Figure 3:**
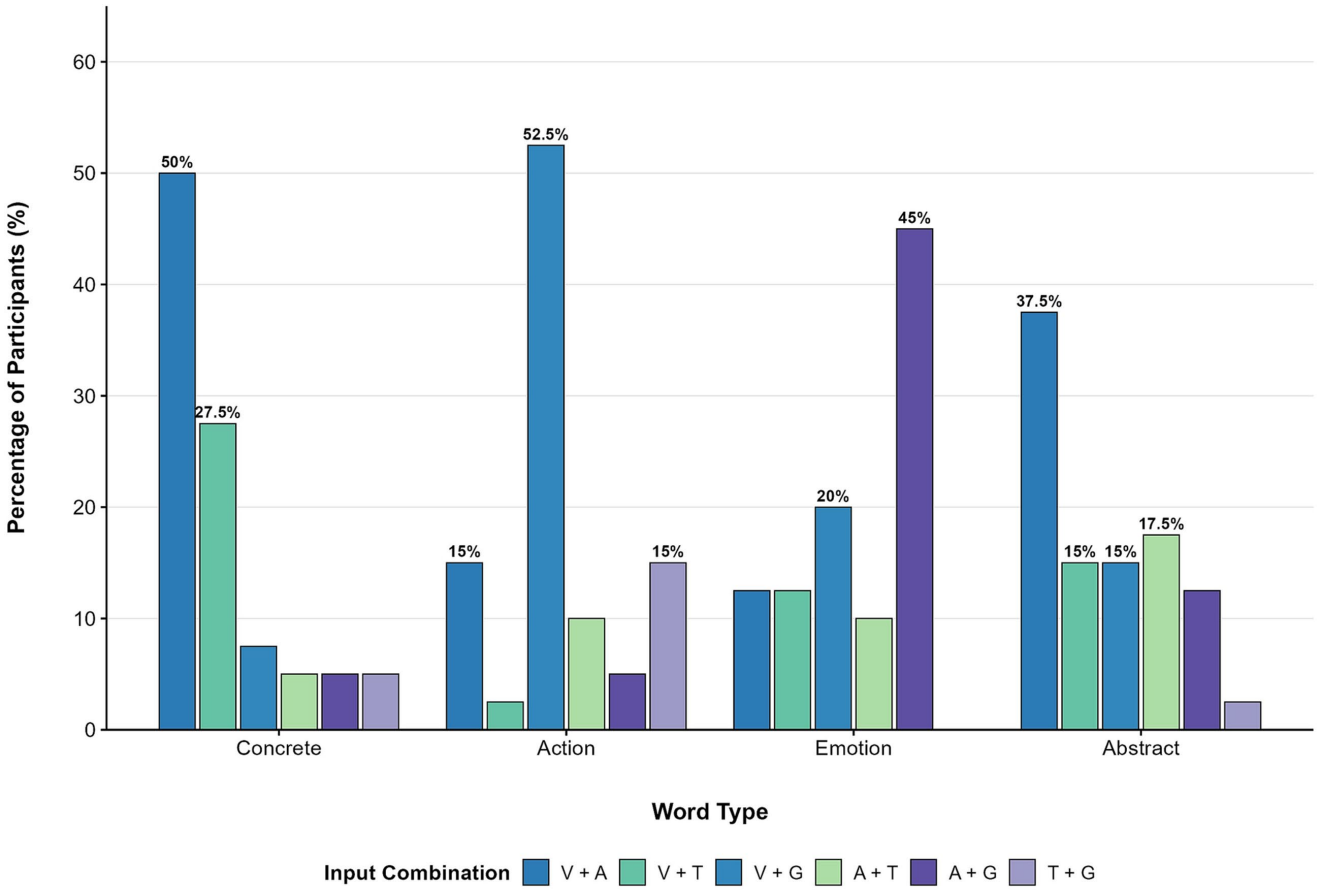
Distribution of learner preferences for input combinations across word types. Learners favored *V + A* for concrete/action words but significantly preferred *A + G* for emotion words.

Participants selected their preferred dual-modality combination for each of four word types (Figure 3). The distribution of combination preferences differed significantly across word types, *χ*^2^ (15) =.

80.48, *p* < 0.001, Cramér’s *V* = 0.41. For concrete words, visual + auditory was most frequently selected (50.0%), followed by visual + tactile (27.5%). For action words, visual + gestural was the dominant choice (52.5%). For emotion words, auditory + gestural was most preferred (45.0%), with visual + gestural second (20.0%). Preferences for abstract words were more dispersed: visual + auditory was selected most often (37.5%), but auditory + tactile (17.5%), visual + gestural (15.0%), and visual + tactile (15.0%) were also selected.

Exploratory analyses examined whether combination preferences varied by proficiency level. Applying Bonferroni correction for multiple comparisons across four word types (*α* = 0.05/4 = 0.0125), none reached statistical significance after correction. For abstract words, the uncorrected chi-square test suggested a potential association, *χ*^2^ (10) = 19.70, *p* = 0.032, corroborated by Fisher’s exact test (*p* = 0.032) and Monte Carlo simulation with 10,000 replications (*P_sim_* = 0.022). Among advanced learners, 80.0% (8 of 10) selected visual + auditory, whereas intermediate learners’ preferences were distributed across visual + auditory (20.0%), auditory + tactile (25.0%), and auditory + gestural (25.0%). However, given small cell sizes and the exploratory nature of this analysis, this pattern should be interpreted cautiously. Combination preferences for the remaining word types showed no association with proficiency (concrete: *P_sim_* = 0.148; action: *P_sim_* = 0.555; emotion: *P_sim_* = 0.064).

The following analyses of input sequence preferences are exploratory in nature and should be interpreted accordingly. The questionnaire also assessed preferences for input presentation order among three modalities (visual, auditory, and gestural). Regarding initial modality, visual was most frequently preferred (52.5%), followed by auditory (15.0%), with 17.5% indicating that their preference depended on word type, *χ*^2^ (4) = 28.25, *p* < 0.001. Preferences for overall trimodal sequence also varied by word type, *χ*^2^ (6) = 19.15, *p* = 0.004, Cramér’s *V* = 0.24. For concrete words, 75.0% of participants preferred visual → auditory → gestural; for action words, 60.0% selected this same sequence. Preferences were more variable for abstract and emotion words. For abstract words, 45.0% preferred auditory → visual → gestural, while 35.0% preferred visual → auditory → gestural. For emotion words, 47.5% selected visual → auditory → gestural, and 30.0% preferred gestural → visual → auditory.

Most participants reported believing that presentation sequence affected vocabulary learning effectiveness, *χ*^2^ (2) = 13.85, *p* < 0.001: 55.0% reported perceiving a large effect, 37.5% a small effect, and 7.5% no effect. These responses differed by proficiency level, *χ*^2^ (4) = 12.44, *p* = 0.011 (Monte Carlo *p*, *B* = 10,000); 30.0% of advanced learners reported that sequence did not matter, whereas no lower-intermediate or intermediate learners selected this option. When asked about learning outcomes when inputs were presented in their preferred order, 52.5% selected “much better” and 42.5% selected “a little better” (*M* = 1.55, *SD* = 0.68; 1 = *much better*, 5 = *much worse*).

## 4 Discussion

A fundamental caveat applies throughout all findings reflect learner perceptions of hypothetical input scenarios, not measured vocabulary acquisition. Research has repeatedly shown that learners sometimes prefer strategies that feel easier yet produce weaker retention—a phenomenon known as desirable difficulties (Bjork and Bjork, 2011; Deslauriers et al., 2019). The present findings should therefore be read as documentation of what learners believe works, not as evidence of what actually works, and require experimental validation before instructional conclusions can be drawn.

Previous research on multimodal vocabulary learning has predominantly examined specific combinations, particularly audiovisual input (Bisson et al., 2014; Yu and Liu, 2022), with limited systematic comparison across broader modal configurations. The present survey study addressed this gap by documenting learners’ perceptions across 14 input combinations, four word types, and three proficiency levels, providing a more comprehensive picture of how learners evaluate multimodal vocabulary input.

### 4.1 Perceived effectiveness and input complexity

Learners’ ratings showed no significant difference across single, dual, and triple combinations (EMMs: 3.94, 3.79, 3.83), suggesting that adding more modalities did not automatically increase perceived effectiveness. This pattern is broadly consistent with Cognitive Load Theory: when multiple input channels compete for limited working memory resources, added complexity may offset potential benefits rather than enhance them (Sweller et al., 2011). Taken together, these findings partially support CLT over DCT in the context of modality quantity: adding channels did not increase perceived effectiveness, consistent with the prediction that working memory constraints limit the benefit of additional input streams (Sweller et al., 2011). DCT’s prediction that dual-channel encoding enhances processing was not reflected in complexity ratings, though the consistently high ratings for visual-inclusive combinations suggest that the *type* of channel matters more than the *number*—a distinction both frameworks can accommodate.

Within this overall pattern, visual input stood out consistently. The three highest-rated combinations—visual only (*M* = 4.40), visual + auditory (*M* = 4.37), and visual + auditory + tactile (*M* = 4.28)—all included a visual component. Gestural input alone received the lowest rating (*M* = 3.37). Dual Coding Theory offers one possible explanation. Visual input may activate the nonverbal imagery system alongside verbal processing, creating two independent retrieval pathways (Paivio and Walsh, 1993). Chinese EFL learners’ instructional background may have reinforced this pattern. Extended experience with reading and handwriting builds familiarity with visual and tactile channels, which may reduce the extraneous load these channels impose (Kalyuga, 2007; Hu, 2002). Whether learners’ ratings reflect genuine encoding benefits or simply greater comfort with familiar modalities cannot be determined from the present data.

Proficiency was also associated with ratings. Advanced learners tended to score combinations higher than intermediate and lower-intermediate learners (*η*^2^ₚ = 0.29), and this difference coincided with variation in perceived processing difficulty. Higher difficulty correlated negatively with perceived effectiveness (*r* = −0.48), which aligns with evidence that cognitive effort can color learners’ evaluations of their own performance (Seufert, 2020). The proficiency effect was even more pronounced for processing difficulty (*η*^2^ₚ = 0.55), where proficiency accounted for over half the variance in how demanding learners found multimodal input—considerably more than input complexity itself (*η*^2^ₚ = 0.01). Together, these effect sizes indicate that learner proficiency accounted for approximately 29–55% of variance in perceptions, compared to only 1% for modality quantity, suggesting that who is learning may matter more than how many channels are presented when it comes to perceived effectiveness and cognitive load. This pattern aligns with CLT’s prediction that schema availability—which increases with proficiency—determines processing efficiency (Kalyuga, 2007), though the cross-sectional design limits causal interpretation.

### 4.2 Word type-specific combination preferences

Learners demonstrated distinct combination preferences across word categories, *χ*^2^ (15) = 80.48, *p* < 0.001, Cramér’s *V* = 0.41. For concrete nouns, visual + auditory was preferred by 50.0% of participants. For action verbs, visual + gestural was the most common choice (52.5%). This pattern is compatible with embodied cognition perspectives linking physical actions with motor simulation (Lu and Yang, 2025; Kosmas and Zaphiris, 2020), though whether learners consciously drew on such principles remains unclear.

For emotion words, auditory + gestural was most preferred (45.0%), possibly because prosodic features naturally convey affective states (Liebenthal et al., 2016) and expressive gestures enhance emotional communication (Aslan et al., 2024).

These preferences may also reflect learners’ instructional background. Chinese EFL classrooms have traditionally emphasized visual and tactile input through reading and handwriting (Hu, 2002; Zhou et al., 2025). Auditory and gestural channels, being less common in this context, may have been perceived as supplementary rather than primary input. Combination preferences documented here may therefore not generalize to learners from other instructional traditions.

These differentiated patterns suggest that learners perceive certain modality pairings as more appropriate for specific semantic categories. While experimental studies have examined how different modalities affect vocabulary acquisition (Farley et al., 2014; Huang et al., 2019; Lin et al., 2016), the present findings complement this work by documenting how learners themselves conceptualize preferred input for different word types. Whether these preferences correspond to actual learning advantages requires experimental validation.

For abstract words, preferences were more dispersed: although visual + auditory was most frequently selected (37.5%), other combinations each received 15–17.5% support. This lack of consensus may reflect the inherent difficulty of grounding abstract concepts in sensory experience (Dove, 2016; Kaushanskaya and Rechtzigel, 2012), and suggests that learners may be less certain about which modality combinations suit this word type.

Beyond combination types, 52.5% of participants preferred visual input as the starting modality, and 92.5% believed that presentation order affected learning outcomes. While this indicates a prevalent perception among learners, interpretation should consider potential demand characteristics (Orne, 1962)—when explicitly asked whether order matters, participants may overestimate its importance. That said, verifying whether these preferences yield actual learning benefits is advisable, given that self-reported perceptions of learning may not always align with actual performance (Deslauriers et al., 2019). Future experimental designs would benefit from systematically comparing different presentation orders while controlling for expectancy effects.

### 4.3 Differences across proficiency levels

RQ3 examined proficiency-related differences in input preferences. As reported above, proficiency was significantly associated with overall perceived effectiveness ratings (*η*^2^ₚ = 0.29), with advanced learners rating input combinations higher than intermediate and lower-intermediate learners. Beyond this general pattern, a significant word type × proficiency interaction emerged, *F*(6, 111.0) = 2.98, *p* = 0.010, *η*^2^ₚ = 0.14, which, by conventional benchmarks (Cohen, 1988), represents a large effect, suggesting that proficiency-specific patterns in word type perceptions are not trivial.

For abstract words, intermediate learners reported the lowest helpfulness ratings (EMM = 3.05), below both lower-intermediate (EMM = 3.90) and advanced learners (EMM = 4.60), forming an unexpected *U*-shaped pattern.

The *U*-shaped pattern across proficiency levels can be interpreted through CLT’s distinction between extraneous and germane cognitive load (Sweller et al., 2011). For lower-intermediate learners, cognitive resources may be largely consumed by basic decoding demands, leaving insufficient capacity to register the particular difficulty abstract words pose for sensory grounding. Intermediate learners, having reduced their extraneous load through accumulated exposure, may become more sensitive to this difficulty—yet lack the developed schemas needed to allocate germane load toward deeper semantic encoding. The result may be a gap between awareness of difficulty and the capacity to address it, reflected in the lowest ratings in the sample. Advanced learners, by contrast, may bring sufficiently elaborated schemas to the task that germane resources can be directed toward meaning construction—for instance, connecting abstract words to concrete anchor examples or generating contextual definitions (Schmitt, 2008)—allowing them to engage more productively with multimodal input regardless of combination type. Given the *post hoc* nature of this interpretation and the small subgroup sizes, however, replication with direct measures of strategy use and schema development remains necessary.

Abstract words were also the only category showing potential proficiency-related differences in combination preferences. Although the association did not survive Bonferroni correction for multiple comparisons (uncorrected *p* = 0.032), converging evidence from chi-square, fisher’s exact, and Monte Carlo tests suggests the pattern merits attention: 80.0% of advanced learners preferred visual + auditory, whereas intermediate learners’ choices were distributed across multiple combinations (visual + auditory: 20.0%; auditory + tactile: 25.0%; auditory + gestural: 25.0%). This suggests that preference consolidation may occur as proficiency increases, at least for abstract vocabulary, and may reflect the emergence of more systematic encoding strategies at higher proficiency levels (Schmitt, 2008) though the small sample size requires cautious interpretation.

### 4.4 Pedagogical implications

The differentiated preferences across word types suggest that modality selection may matter more than modality quantity. Rather than uniformly increasing input channels, materials could be tailored to word characteristics: pairing images with narrated sentence contexts for concrete nouns, incorporating gestural demonstrations such as physically enacting *collapse* or *grasp* for action verbs, and using audio recordings that model emotional prosody for emotion words. The comparable perceived effectiveness ratings across single, dual, and triple combinations—alongside visual-only input achieving the highest mean rating (*M* = 4.40)—further suggest that a carefully designed single-modality resource may serve learners better than a hastily assembled multimodal alternative, which carries practical implications for resource-constrained settings. These suggestions remain grounded in learner perceptions rather than performance data and should be treated as hypotheses awaiting experimental confirmation.

The proficiency-related findings for abstract vocabulary point to a more specific instructional need. Intermediate learners showed the most dispersed combination preferences and the lowest perceived effectiveness ratings for this word type (EMM = 3.05), suggesting that varying modality alone may be insufficient. Instructors might consider making encoding strategies explicit—guiding learners to construct mental images, link abstract words to concrete anchor examples, or practice verbal elaboration by generating their own definitions or example sentences (Schmitt, 2008). For presentation sequences, the strong preference for visual-first input (52.5%) offers a tentative starting point, consistent with evidence that visual input may support subsequent linguistic encoding (Yu and Liu, 2022), though instructors should remain cautious about rigid sequencing given that 30% of advanced learners reported order made no difference.

## 5 Limitations

This study has several limitations. The sample was small (*N* = 40) and unevenly distributed across proficiency levels (10 lower-intermediate, 20 intermediate, 10 advanced), which limited power for detecting interactions. Some patterns that appeared potentially meaningful—such as how advanced learners preferred visual + auditory for abstract words while intermediate learners’ choices dispersed—did not survive Bonferroni correction and require replication with larger samples. The sample also skewed heavily female (90%), so we cannot determine whether gender affects modality preferences.

The design introduced additional constraints. Because all data came from self-reports about hypothetical scenarios, we cannot verify whether perceived effectiveness aligns with actual learning or whether reported processing difficulty reflects genuine cognitive load. The questionnaire’s explicit questions about presentation sequence may have inflated its perceived importance through demand characteristics. Additionally, our operational definition of “tactile input” combined distinct activities—handling flashcards versus writing/copying—which may involve different cognitive processes even though both engage the hands. The absence of formal pilot testing also limits available evidence for the questionnaire’s construct validity.

These constraints point to clear next steps. Experimental studies comparing retention across modality combinations would reveal whether preferences translate into learning advantages. Such experiments could also test whether sequence actually matters by manipulating order while controlling for expectancy effects. Tracking learners longitudinally would show whether preferences shift as proficiency develops, and adding objective measures like eye-tracking could clarify what drives modality choices.

The present findings nonetheless document systematic patterns in how Chinese EFL learners perceive multimodal vocabulary input. Learners distinguish among modalities based on word type and judge specific combinations rather than sheer quantity of input channels. These perceptions vary with proficiency in ways that suggest developmental changes in how learners approach vocabulary learning. Whether these patterns predict actual learning outcomes remains an open question for experimental research.

## Data Availability

The original contributions presented in the study are included in the article/Supplementary material, further inquiries can be directed to the corresponding authors.
