# Supplementary material for: Multimodal input for vocabulary learning: Chinese EFL learners’ perceived effectiveness across input combinations, word types, and proficiency levels
Source: Front Psychol. 2026 Mar 23;17:1783303. doi: 10.3389/fpsyg.2026.1783303 (PMC13050825; doi:10.3389/fpsyg.2026.1783303)
Supplement: Supplementary file 1 [file Data_sheet_1.zip › Supplementary figure 3.pdf]

Supplementary figure 3

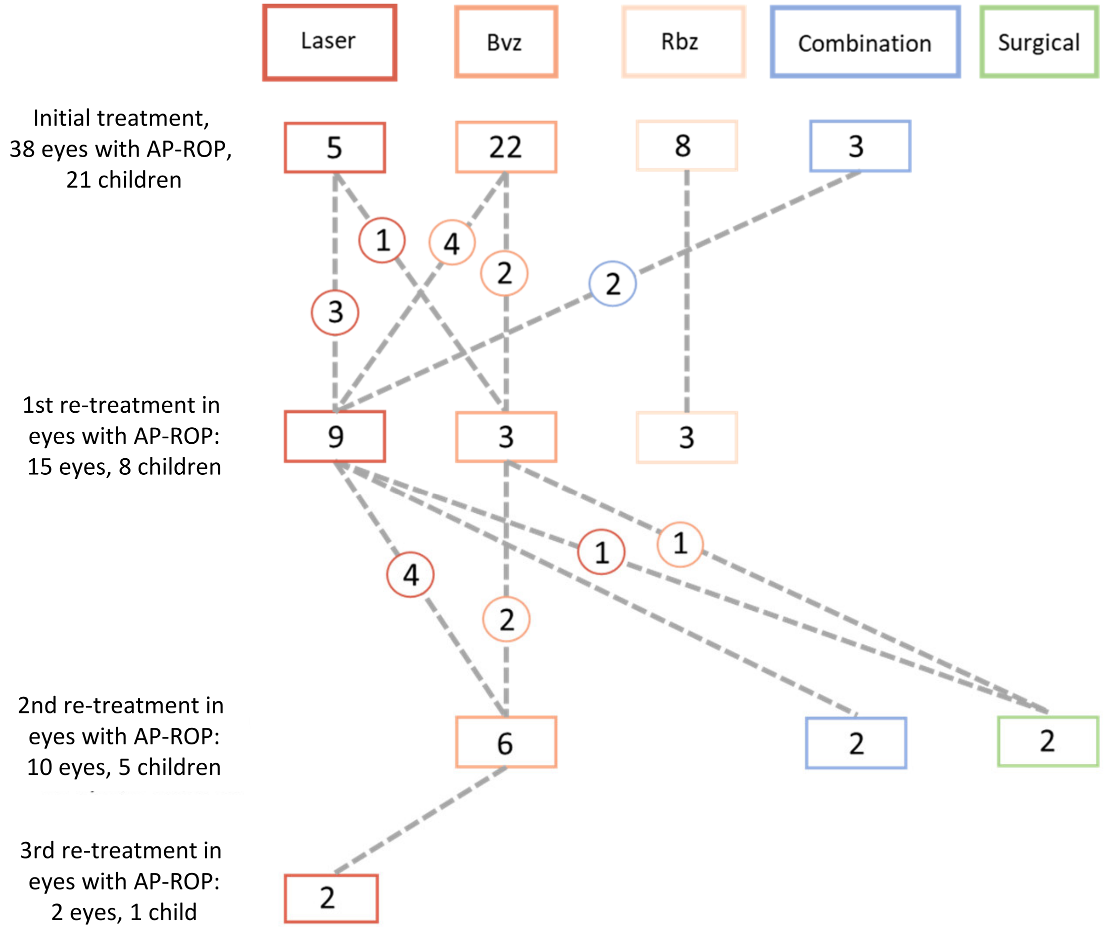

Retreatment pattern for 38 eyes with AP-ROP at initial treatment. 15 eyes of 38 eyes needed a first retreatment. 10 eyes a second retreatment and 2 eyes even a third retreatment.
