# Supplementary material for: Multimodal input for vocabulary learning: Chinese EFL learners’ perceived effectiveness across input combinations, word types, and proficiency levels
Source: Front Psychol. 2026 Mar 23;17:1783303. doi: 10.3389/fpsyg.2026.1783303 (PMC13050825; doi:10.3389/fpsyg.2026.1783303)
Supplement: Supplementary file 1 [file Data_sheet_1.zip › Supplementary figure 4.pdf]

Supplementary figure 4

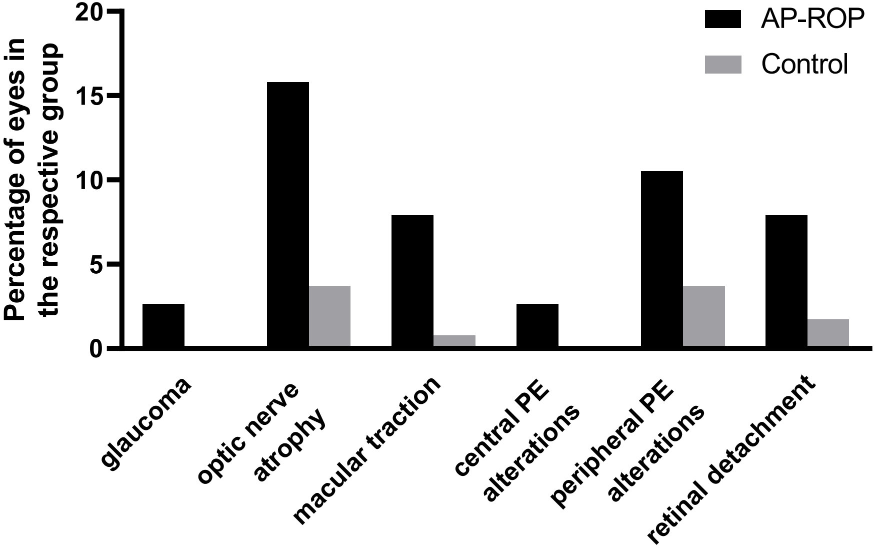

Distribution of ocular findings (glaucoma, optic nerve atrophy, macular traction, central pigment epithelial (PE) alterations, peripheral pigment epithelial alterations, retinal detachment) in the AP-ROP and control group. More ocular findings were documented in the AP-ROP group.
