# Supplementary material for: Multimodal input for vocabulary learning: Chinese EFL learners’ perceived effectiveness across input combinations, word types, and proficiency levels
Source: Front Psychol. 2026 Mar 23;17:1783303. doi: 10.3389/fpsyg.2026.1783303 (PMC13050825; doi:10.3389/fpsyg.2026.1783303)
Supplement: Supplementary file 1 [file Data_sheet_1.zip › Appendix 1.docx]

**Retina.net ROP Registry Study Group (in alphabetical order):**

Sabine Aisenbrey, Department of Ophthalmology, Vivantes Klinikum Neukoelln, Berlin, Germany

Tarek Aljarmakani, Department of Ophthalmology, University Medical Center Goettingen, Goettingen, Germany

Matthais Altmann, Department of Ophthalmology, University of Regensburg, Regensburg, Germany

Monika Andrassi-Darida, Department of Ophthalmology, Justus-Liebig-University Giessen, Eye Clinic, University Hospital Giessen and Marburg GmbH, Campus Giessen, Germany

Anna Bajor, University Eye Hospital, Hannover Medical School, Hannover, Germany

Sabine Baumgarten, Department of Ophthalmology, University Hospital RWTH Aachen, Germany

Teresa Barth, Department of Ophthalmology, University of Regensburg, Regensburg, Germany

Viktorija Belousova, Department of Ophthalmology, University of Luebeck, Luebeck, Germany

Sebastian Bemme, Department of Ophthalmology, University Medical Center Goettingen, Goettingen, Germany

Tim Bleul, Eye Center, Medical Center, Faculty of Medicine, University of Freiburg, Freiburg, Germany

Benedikt Blüml, Department of Ophthalmology, University of Regensburg, Regensburg, Germany

Bettina Bohnhorst, University Eye Hospital, Hannover Medical School, Hannover, Germany

Caroline Böhne, University Eye Hospital, Hannover Medical School, Hannover, Germany

Maximilian Busch, Department of Ophthalmology, University of Luebeck, Luebeck, Germany

Helge Breuß, Department of Ophthalmology, HELIOS Klinikum Berlin-Buch, Berlin, Germany

Dorothee Brockmann, Department of Neonatology, Hannover Medical School, Hannover, Germany

Marie-Christine Bründer, Department of Ophthalmology, University Medicine Greifswald, Greifswald, Germany

Alexandra T. Camp, Eye Center, Medical Center, Faculty of Medicine, University of Freiburg, Freiburg, Germany

Lars Choritz, Department of Ophthalmology, Otto von Guericke University, Magdeburg, Germany

Meltem Elcivan, University Eye Hospital, Hannover Medical School, Hannover, Germany

Verena Englmaier, Department of Ophthalmology, University of Muenster Medical Center, Muenster, Germany

Nicole Eter, Department of Ophthalmology, University of Muenster Medical Center, Muenster, Germany

Silvia Falkenau, Department of Ophthalmology, University of Muenster Medical Center, Muenster, Germany

Navid Farassat, Eye Center, Medical Center, Faculty of Medicine, University of Freiburg, Freiburg, Germany

Olga Furashova, Department of Ophthalmology, Klinikum Chemnitz gGmbH, Chemnitz, Germany

Carsten Framme, University Eye Hospital, Hannover Medical School, Hannover, Germany

Ameli Gabel-Pfisterer, Department of Ophthalmology, Klinikum Ernst von Bergmann, Potsdam, Germany

Barbara Glitz, Department of Ophthalmology, University of Muenster Medical Center, Muenster, Germany

Stefanie Gniesmer, Department of Ophthalmology, University of Luebeck, Luebeck, Germany

Salvatore Grisanti, Department of Ophthalmology, University of Luebeck, Luebeck, Germany

Thomas Gröber, University Eye Hospital, Hannover Medical School, Hannover, Germany

Rainer Guthoff, Department of Ophthalmology, Faculty of Medicine, University of Duesseldorf, Duesseldorf, Germany

Nicolai Gross, Eye Center, Medical Center, Faculty of Medicine, University of Freiburg, Freiburg, Germany

Horst Helbig, Department of Ophthalmology, University of Regensburg, Regensburg, Germany

Jonas Herden, University Eye Hospital, Hannover Medical School, Hannover, Germany

Karsten Hufendiek, University Eye Hospital, Hannover Medical School, Hannover, Germany

Christoph Jacobi, University Eye Hospital, Hannover Medical School, Hannover, Germany

Christina Jacobsen, University Eye Hospital, Hannover Medical School, Hannover, Germany

Herbert Jägle, Department of Ophthalmology, University of Regensburg, Regensburg, Germany

Lutz Joachimsen, Eye Center, Medical Center, Faculty of Medicine, University of Freiburg, Freiburg, Germany

Vinodh Kakkassery, Department of Ophthalmology, Klinikum Chemnitz gGmbH, Chemnitz, Germany

Sema Kaya, Department of Ophthalmology, Faculty of Medicine, University of Duesseldorf, Duesseldorf, Germany

Annette Keller-Wackerbauer, Department of Neonatology, Barmherzige Brüder Hospital Regensburg, Germany

Ala Khamees, Department of Ophthalmology, Faculty of Medicine, University of Duesseldorf, Duesseldorf, Germany

Tim U. Krohne, Department of Ophthalmology, Faculty of Medicine and University Hospital Cologne, University of Cologne, Cologne, Germany

Sandra Kroll, Department of Ophthalmology, Klinikum Ernst von Bergmann, Potsdam, Germany

Sebastian Küchlin, Eye Center, Medical Center, Faculty of Medicine, University of Freiburg, Freiburg, Germany

Wolf A. Lagrèze, Eye Center, Medical Center, Faculty of Medicine, University of Freiburg, Freiburg, Germany

Jeany Q. Lammert, Department of Ophthalmology, Faculty of Medicine and University Hospital Cologne, University of Cologne, Cologne, Germany

Florian Langhammer, Department of Neonatology, Barmherzige Brüder Hospital Regensburg, Germany

Marian Liegl, Department of Ophthalmology, Vivantes Klinikum Neukoelln, Berlin, Germany

Raffael Liegl, Department of Ophthalmology, University Medicine Bonn, Bonn, Germany

Lyubomyr Lytcynchuk, Department of Ophthalmology, Justus-Liebig-University Giessen, Eye Clinic, University Hospital Giessen and Marburg GmbH, Campus Giessen, Germany

Nils Mester, University Eye Hospital, Hannover Medical School, Hannover, Germany

Emilia Michalewicz, Department of Ophthalmology, University Hospital RWTH Aachen, Germany

Holger Michel, Department of Neonatology, Barmherzige Brüder Hospital Regensburg, Germany

Melanie Michelczak, Department of Ophthalmology, Vivantes Klinikum Neukoelln, Berlin, Germany

Martina Möglich, Department of Ophthalmology, Klinikum Ernst von Bergmann, Potsdam, Germany

Fanni Molnar, Eye Center, Medical Center, Faculty of Medicine, University of Freiburg, Freiburg, Germany

Franziska Mühmel, Department of Ophthalmology, Klinikum Chemnitz gGmbH, Chemnitz, Germany

Anna Nguyen-Höhl, Department of Ophthalmology, University Medical Center Goettingen, Goettingen, Germany

Justus Obergassel, Department of Ophthalmology, University of Muenster Medical Center, Muenster, Germany

Laurenz Johannes Bernhard Pauleikhoff, Department of Ophthalmology, University Medical Center Hamburg Eppendorf, Hamburg, Germany

Corinna Peter, University Eye Hospital, Hannover Medical School, Hannover, Germany

Johanna M. Pfeil, Department of Ophthalmology, University Medicine Greifswald, Greifswald, Germany

Amelie Pielen, University Eye Hospital, Hannover Medical School, Hannover, Germany

Sabine Pirr, University Eye Hospital, Hannover Medical School, Hannover, Germany

Jenny Potratz, Department of Ophthalmology, University of Muenster Medical Center, Muenster, Germany

Julia Maria Pulst Caliman, Department of Ophthalmology, Klinikum Ernst von Bergmann, Potsdam, Germany

Tjark Rauscher, University Eye Hospital, Hannover Medical School, Hannover, Germany

Alena Richter, University Eye Hospital, Hannover Medical School, Hannover, Germany

Michael Reich, Eye Center, Medical Center, Faculty of Medicine, University of Freiburg, Freiburg, Germany

Anne-Kathrin Retzlaff, Department of Neonatology, HELIOS Klinikum Berlin-Buch, Berlin, Germany

Benedikt Rössler, University Eye Hospital, Eberhard Karls University of Tuebingen, Tuebingen, Germany

Julius Caspar Rotering, Department of Ophthalmology, University of Luebeck, Luebeck, Germany

Julia Sandkötter, Department of Ophthalmology, University of Muenster Medical Center, Muenster, Germany

Marlene Sassmannshausen, Department of Ophthalmology, University Medicine Bonn, Bonn, Germany

Christian Schiemenz, Department of Ophthalmology, University of Luebeck, Luebeck, Germany

Verena Schöneberger, Department of Ophthalmology, Faculty of Medicine, University of Duesseldorf, Duesseldorf, Germany

Konrad Schulze, Department of Ophthalmology, University Medicine Greifswald, Greifswald, Germany.

Verena Schwering, Department of Ophthalmology, University of Muenster Medical Center, Muenster, Germany

Anne Schwietering, Eye Center, Medical Center, Faculty of Medicine, University of Freiburg, Freiburg, Germany

Christos Skevas, Department of Ophthalmology, University Medical Center Hamburg Eppendorf, Hamburg, Germany

Martin Spitzer, Department of Ophthalmology, University Medical Center Hamburg Eppendorf, Hamburg, Germany

Andreas Stahl, Department of Ophthalmology, University Medicine Greifswald, Greifswald, Germany.

Jens Storp, Department of Ophthalmology, University of Muenster Medical Center, Muenster, Germany

Patrick Strassburger, Department of Ophthalmology, Klinikum Chemnitz gGmbH, Chemnitz, Germany

Daniela Suesskind, University Eye Hospital, Eberhard Karls University of Tuebingen, Tuebingen, Germany

Maria Tekaat, Department of Ophthalmology, University of Muenster Medical Center, Muenster, Germany

Michael Völker, University Eye Hospital, Eberhard Karls University of Tuebingen, Tuebingen, Germany

Fabienne Wähler, Department of Ophthalmology, University Medicine Greifswald, Greifswald, Germany

Peter Walter, Department of Ophthalmology, University Hospital RWTH Aachen, Germany

Sven Wellmann, Department of Neonatology, Barmherzige Brüder Hospital Regensburg, Germany
