# Supplementary material for: Multimodal input for vocabulary learning: Chinese EFL learners’ perceived effectiveness across input combinations, word types, and proficiency levels
Source: Front Psychol. 2026 Mar 23;17:1783303. doi: 10.3389/fpsyg.2026.1783303 (PMC13050825; doi:10.3389/fpsyg.2026.1783303)
Supplement: Supplementary file 1 [file Data_sheet_1.zip › Suppelementary table 2.pdf]

Supplementary Table 2. Functional outcome data at follow-up visits of infants with AP-ROP

	initial treatment	retreat-ment#	PNA (months)	Fixation RE	Fixation LE	Strabismus RE	Strabismus LE	Right Eye Sphere	Right Eye Cylinder	Right Eye Spherical Equivalent	Right Eye Axis	Left eye Sphere	Left eye Cylinder	Left eye Spherical Equivalent	Left eye Axis	Comment
1	bil laser and bvz	bil	11.4*	central	central	exotropia, intermittent	no strabismus	4	-1	3,50	10	1	-1	0,50	10	RE: severe macular traction and complete retinal detachment
			14.9	central		exotropia, intermittent	no strabismus									RE: severe makular traction and complete retinal detachment
			31.0		central	exotropia, continuous	no strabismus									both eyes severe macular traction RE: complete retinal detachment and moderate glaucoma
2	bil laser	bil	5.8	central	central	no strabismus	no strabismus	0	0	0,00	0	0	0	0,00	0	LE: mild partial retinal detachment
			14.1*	central	excentrical	no strabismus	exotropia, continuous	-1	0	-1,00	0	0	0	0,00	0	LE: mild partial retinal detachment
			34.4	central	excentrical	no strabismus	exotropia, continuous	-3	-1	-3,50	50	-3	0	-3,00	0	LE: mild partial retinal detachment
3	bil bvz	bil	7.9*					0	0	0,00	0	0	0	0,00	0	BE: peripheral pigment epithelial alterations
			17.3	central	central	no strabismus	no strabismus	0	0	0,00	0	0	0	0,00	0	
			22.1	central	central	esotropia, continuous	no strabismus	0	0	0,00	0	0	0	0,00	0	
			28.6	central	central	esotropia, intermittent	esotropia, intermittent	0	0	0,00	0	0	0	0,00	0	
			75.5	central	central	esotropia, intermittent	esotropia, intermittent	-7	-4	-9,00	75	-7	-3	-8,50	100	BE: moderate optic nerve atrophy
4	bil bvz	no	8.5*	central	central	esotropia, intermittent	esotropia, intermittent									
5	bil rbz	no	8.5*	*central*	central	*no strabismus*	no strabismus	*2*	*-1*	*1,50*	*170*	1	-1	0,50	180	
			15.9	*excentrical*	central	*esotropia, continuous*	no strabismus	*-1*	*-2*	*-2,00*	*180*	-1	-2	-2,00	180	
			28.0	*excentrical*	central	*esotropia, continuous*	no strabismus	*-2*	*-2*	*-3,00*	*180*	-2	-2	-3,00	180	
6	RE: laser LE: rbz	no	10.8*	central	central	exotropia, intermittent	no strabismus	-7	-2	-8,00	151	-7	0	-7,00	0	RE: moderate macular traction, moderate peripheral pigment endothelial alterations (laser scars)
			25.5	central	central	exotropia, continuous	no strabismus	-1	-2	-2,00	90	0	-1	-0,50	60	RE: moderate macular traction, moderate peripheral pigment endothelial alterations (laser scars)
			41.5	excentrical	central	exotropia, continuous	no strabismus	-5	-1	-5,50	30	-2	-2	-3,00	80	RE: moderate macular traction, moderate peripheral pigment endothelial alterations (laser scars)
			60.8	excentrical	central	exotropia, continuous	no strabismus	-1	-3	-2,50	150	-1	-2	-2,00	90	RE: moderate macular traction, moderate peripheral pigment endothelial alterations (laser scars)
			66.6	excentrical	central	exotropia, continuous	no strabismus	-1	-3	-2,50	150	-1	-2	-2,00	90	RE: moderate macular traction, moderate peripheral pigment endothelial alterations (laser scars)
7	bil bvz	no	14.9*	excentrical	excentrical			18	0	18,00	0	15	0	15,00	0	BE: mild optic nerve atrophy RE: mild macular traction, moderate central pigment epithelial alterations LE: moderate peripheral pigment epithelial alterations
			26.6													aphakia
			40.0													severe visual impairment (possibly blindness)
8	bil laser	bil	23.7			esotropia	esotropia									LE: severe complete retinal detachment
			32.0			esotropia										
9	bil bvz	bil	10.3*	central	central	no strabismus	esotropia, intermittent	-2	-2	-3,00	164	-1	-1	-1,50	180	
10	bil bvz	no	11.0*			no strabismus	esotropia					2	-2	1,00		
			25.0			no strabismus	esotropia	1	1	1,50	4	2	-1	1,50	36	BE: mild optic nerve atrophy
			30.7			no strabismus	esotropia									BE: mild optic nerve atrophy
			50.4			no strabismus	esotropia	1	-1	0,50	0	1	-1	0,50	0	BE: mild optic nerve atrophy
11	bil rbz	bil	9.2*	*central*	central	*no strabismus*	no strabismus	*-4*	*-1*	*-4,50*	*49*	-5	-2	-6,00	109	
12	bil rbz	no	11.1*	central	*central*	no strabismus	*no strabismus*	0	2	1,00	74	*0*	*2*	*1,00*	*98*	

In child 5 and 11 the right eye, in child 12 the left eye had initially been treated due to another stage of ROP not AP-ROP (marked in italics)

#at least one retreatment; RE = right eye; LE = left eye; BE = both eyes; bil=bilateral; bzv=bevacizumab; rbz=ranibizumab

*data from this follow-up visit was used to calculate median spherical equivalent at about 1 year of age.
