# Supplementary material for: Multimodal input for vocabulary learning: Chinese EFL learners’ perceived effectiveness across input combinations, word types, and proficiency levels
Source: Front Psychol. 2026 Mar 23;17:1783303. doi: 10.3389/fpsyg.2026.1783303 (PMC13050825; doi:10.3389/fpsyg.2026.1783303)
Supplement: Supplementary file 1 [file Data_sheet_1.zip › Supplementary figure 1.pdf]

Supplementary figure 1

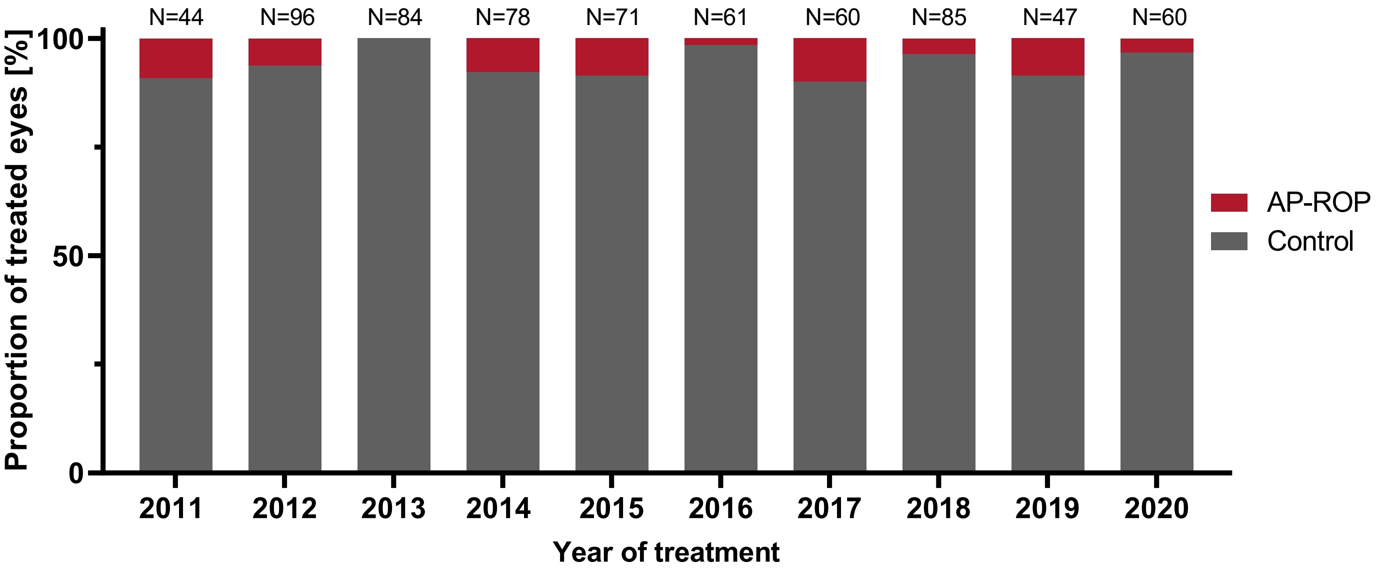

Development of proportion of eyes with AP-ROP during observation period. No trend for a general increase of decrese in the proportion of AP-ROP was observed.
