# Supplementary material for: Multimodal input for vocabulary learning: Chinese EFL learners’ perceived effectiveness across input combinations, word types, and proficiency levels
Source: Front Psychol. 2026 Mar 23;17:1783303. doi: 10.3389/fpsyg.2026.1783303 (PMC13050825; doi:10.3389/fpsyg.2026.1783303)
Supplement: Supplementary file 3 [file Data_sheet_3.pdf]

Waipapa Taumata Rau University of Auckland

Welcome Page

Thank you for participating in this research study!

This questionnaire is designed to explore how different types of multimodal input (such as visual, audio, or touch-based input) affect vocabulary learning.

Your answers will help us understand which learning methods are more effective for different learners.

All responses are anonymous and will only be used for academic research purposes.

Part I: Basic Information

What is your age range?

- ○ 18–25
- ○ 26-30
- ○ 31-35
- ○ 36 or above

What is your gender?

- ○ Male
- ○ Female
- ○ Prefer not to say

What is your native language?

- ○ Chinese
- ○ English
- ○ ________ Other (please specify)

What is your English level?

(Based on your latest test, such as IELTS, or self-assessment)

- ○ Beginner (IELTS below 6)
- ○ Intermediate (IELTS 6.5–7.5)
- ○ Advanced (IELTS 8–9)

How long have you learned English?

- ○ Less than 1 year
- ○ 1–3 years
- ○ 3–5 years
- ○ More than 5 years

Which of these have you used when learning English words?
(Select all that apply)

- ☐ Reading or looking at pictures (visual)
- ☐ Listening to the word (auditory)
- ☐ Touching or using flashcards (tactile)
- ☐ Using gestures (gestural)

Block 1

Part II: Multimodal Input Preferences

How effective are the following input types for your vocabulary learning?
(1 = Not effective at all, 5 = Very effective)

	1 (Not effective at all)	2	3	4	5 (Very effective)
**Visual** *(e.g., text, images, videos)*	○	○	○	○	○
**Auditory** *(e.g., pronunciation, audio)*	○	○	○	○	○
**Tactile** *(e.g., using flashcards, making vocabulary posters)*	○	○	○	○	○
**Gestural** *(e.g., hand movements, role-play with gestures)*	○	○	○	○	○

Which input types do you most enjoy using when learning new words? (Select 1-3)

- ☐ Visual *(e.g., reading a word in a textbook or on a flashcard)*
- ☐ Auditory *(e.g., listening to a word's pronunciation in an audio clip)*
- ☐ Tactile *(e.g., touching something cold to learn the word "cold")*
- ☐ Gestural *(e.g., acting out the word "run" with your hands)*

Which input types feel most difficult or tiring when learning new words? (Select 1-3)

- ☐ Visual *(e.g., reading a word in a textbook or on a flashcard)*
- ☐ Auditory *(e.g., listening to a word's pronunciation in an audio clip)*
- ☐ Tactile *(e.g., touching something cold to learn the word "cold")*
- ☐ Gestural *(e.g., acting out the word "run" with your body or hands)*

How effective are the following two-input combinations for your vocabulary learning?

(1 = Not effective at all, 5 = Very effective)

	1 (Not effective at all)	2	3	4	5 (Very effective)
**Visual + Auditory** *(e.g., watching a video with sound)*	○	○	○	○	○
**Visual + Gestural** *(e.g., seeing "clap" and clapping)*	○	○	○	○	○
**Visual + Tactile** *(e.g., copying a word from a flashcard)*	○	○	○	○	○
**Auditory + Tactile** *(e.g., touching an item you hear)*	○	○	○	○	○
**Auditory + Gestural** *(e.g., hearing "jump" and jumping)*	○	○	○	○	○
**Tactile + Gestural** *(e.g., flipping a flashcard and doing a gesture)*	○	○	○	○	○

Is using three input types more effective than using two when learning vocabulary?

- ○ Yes
- ○ About the same
- ○ No

Rate how helpful multiple input types are in these learning situations:

(1 = Not helpful, 5 = Very helpful)

	1	2	3	4	5
During formal classes	○	○	○	○	○
When studying alone	○	○	○	○	○
When preparing for tests	○	○	○	○	○

Block 2

Part III: Processing Challenges and Cognitive Load

When using multiple input types together, how often do you experience these problems?

(1 = Never, 5 = Always)

	1 (Never)	2	3	4	5 (Always)
Too much information at once	○	○	○	○	○
Need more time to process information	○	○	○	○	○
Hard to focus on what's important	○	○	○	○	○

	1 (Never)	2	3	4	5 (Always)
Different inputs give confusing information	○	○	○	○	○
Feel mentally tired	○	○	○	○	○

How helpful are these THREE-input combinations? (1 = Not helpful, 5 = Very helpful)
(See = visual, Hear = auditory, Write = tactile, Act = gestural)

	1	2	3	4	5
See + Hear + Write					
See + Hear + Act					
See + Write + Act					
Hear + Write + Act					

How tired do you feel when using three input types compared to two?

- ○ Not tired at all
- ○ Slightly tired
- ○ Moderately tired
- ○ Very tired

When learning with multiple input types, I feel that:

- ○ Easy and helpful
- ○ Mostly fine, but sometimes too much
- ○ Manageable with effort
- ○ Hard to manage

How many inputs can you handle at once?

- ○ 1 (one only)
- ○ 2 (two together)
- ○ 3 (three together)
- ○ 4 (all four together)
- ○ Depends on the combination

Block 6

Take a Short Break
You're almost finished with the survey
Feel free to take a short break before continuing
Click “Next” when you're ready to go on.

Block 3

Part IV: Wordtypes and Input Combinations

How helpful are multiple inputs for learning these word types?
(1 = Not helpful, 5 = Very helpful)

	1	2	3	4	5
Concrete words (apple\car\book)					
Abstract words (freedom\theory\peace)					
Action words (run\jump\swim)					
Emotion words (angry\happy\excited)					

Which word types are hardest to learn with multiple inputs? (Rank 1-4, 1 = hardest)

Concrete words

Action words

Abstract words

Emotion words

For concrete words (e.g., "car"), which input combination helps you most?

- Visual + Auditory (e.g., seeing a picture and hearing the word)
- Visual + Tactile (e.g., seeing a picture and touching something)
- Visual + Gestural (e.g., seeing a picture and acting it out)
- Auditory + Tactile (e.g., hearing the word and writing it)
- Auditory + Gestural (e.g., hearing the word and showing the emotion)
- Tactile + Gestural (e.g., writing the word and acting it out)

For abstract words (e.g., "success"), which input combination helps you most?

- Visual + Auditory (e.g., seeing a picture and hearing the word)
- Visual + Tactile (e.g., seeing a picture and touching something)
- Visual + Gestural (e.g., seeing a picture and acting it out)
- Auditory + Tactile (e.g., hearing the word and writing it)
- Auditory + Gestural (e.g., hearing the word and showing the emotion)
- Tactile + Gestural (e.g., writing the word and acting it out)

For action words (e.g., "jump"), which input combination helps you most?

- Visual + Auditory (e.g., seeing a picture and hearing the word)
- Visual + Tactile (e.g., seeing a picture and touching something)
- Visual + Gestural (e.g., seeing a picture and acting it out)
- Auditory + Tactile (e.g., hearing the word and writing it)

- Auditory + Gestural (e.g., hearing the word and showing the emotion)
- Tactile + Gestural (e.g., writing the word and acting it out)

For emotion words (e.g., "excited"), which input combination helps you most?

- Visual + Auditory (e.g., seeing a picture and hearing the word)
- Visual + Tactile (e.g., seeing a picture and touching something)
- Visual + Gestural (e.g., seeing a picture and acting it out)
- Auditory + Tactile (e.g., hearing the word and writing it)
- Auditory + Gestural (e.g., hearing the word and showing the emotion)
- Tactile + Gestural (e.g., writing the word and acting it out)

Block 4

Part V: Input Sequence and Timing

When learning new words with different inputs, which one do you prefer to start with?

- Visual (e.g., picture, written word)
- Auditory (e.g., sound, pronunciation)
- Tactile (e.g., touching)
- Gestural (e.g., acting the meaning)
- Depends on the word type

For "elephant" (a concrete word), which input order helps you most?

- **See picture → Hear word → Make gesture**
  (visual → auditory → gestural)
- **Hear word → See picture → Make gesture**
  (auditory → visual → gestural)
- **Make gesture → See picture → Hear word**
  (gestural → visual → auditory)

For "success" (an abstract word), which input order helps you most?

- **See definition → Hear word → Show emotion**
  (visual → auditory → gestural)
- **Hear word → See definition → Show emotion**
  (auditory → visual → gestural)
- **Show emotion → See definition → Hear word**
  (gestural → visual → auditory)

For "jump" (an action word), which input order helps you most?

- **See word → Hear word → Do action**
  (visual → auditory → gestural)
- **Hear word → See word → Do action**
  (auditory → visual → gestural)

- **Do action → See word → Hear word**
  (gestural → visual → auditory)

For “excited” (an emotion word), which input order helps you most?

- **See word → Hear word → Show emotion**
  (visual → auditory → gestural)
- **Hear word → See word → Show emotion**
  (auditory → visual → gestural)
- **Show emotion → See word → Hear word**
  (gestural → visual → auditory)

When inputs come in my preferred order, my learning is:

- Much better
- A little better
- No effect
- A little worse
- Much worse

Overall, do you think input sequence (order) affects your vocabulary learning?

- Yes, sequence makes a big difference
- Yes, but the effect is small
- No, sequence doesn't matter

Block 5

Thank you for your participation!

Your responses are valuable to our research on multimodal input in vocabulary learning.

If you have any questions about this study or wish to withdraw your responses, please **save your submission ID** and contact the researcher.

Your participation in this research is greatly appreciated!

Please click “Next” to submit your responses.

Powered by Qualtrics
